# Analysis of the singing voice handicap index for elderly choristers

**DOI:** 10.1590/2317-1782/20212020302

**Published:** 2022-10-25

**Authors:** Weluma Evelyn Rodrigues Moura, Camila Dalbosco Gadenz, Isadora de Oliveira Lemos, Ângelo José Gonçalves Bós, Mauriceia Cassol

**Affiliations:** 1 Departamento de Fonoaudiologia, Universidade Federal de Ciências da Saúde – UFCSPA - Porto Alegre (RS), Brasil.; 2 Departamento de Medicina, Pontifícia Universidade Católica do Rio Grande do Sul – PUCRS - Porto Alegre (RS), Brasil.

**Keywords:** Voice, Voice Quality, Singing, Aged, Quality of Life

## Abstract

**Purpose:**

To analyze the singing voice handicap index in elderly choristers and verify its relationship with the profile, habits and health conditions of the participants.

**Methods:**

110 individuals aged 60 years or older, participating in amateur choirs, were included. Choir singers were interviewed in order to verify data such as age, time in choir singing, vocal classification, and the presence of habits and health conditions adverse to voice production. Subsequently, they answered the questionnaire “Singing Voice Handicap Index (SVHI)”, which assesses the individual's self-perception in relation to experiences in the use of the singing voice.

**Results:**

The SVHI score had a median of 25, with a minimum score of 0 and a maximum score of 86. The most scored items were related to physical aspects in the use of singing voice: “I am unable to use my ‘high voice’” (Q10) and “My throat is dry when I sing”(Q13). It was found that older adults over 75 years of age had a greater voice handicap when compared to younger ones (p=0.020). Choir singers classified as contralto also had a higher SVHI score (p=0.023), as well as individuals who reported drinking little water (p=0.007).

**Conclusion:**

The choristers in this study presented a singing voice handicap index compatible with healthy singing voices. When verifying the relationship of the SVHI score with the characteristics of the participants and with respect to habits and health conditions, it was found that the elderly choir singers over 75 years old, the contralto choir singers, and those who claimed to drink little water had higher scores for the singing voice handicap.

## INTRODUCTION

Voice changes can appear at any age for different reasons, however, during aging, there is a natural and gradual process of loss of vocal efficiency known as presbyphonia. Common symptoms associated are breathiness, hoarseness, loudness changes, the increase in pitch for men and decrease for women, instability, and greater effort during phonation^(1-3)^.

The vocal changes resulting from aging are, however, more subtle in individuals who sing, since frequent singing acts as a moderator of the negative effects related to this process^(4)^. When comparing the voices of elderly singers and non-singers, studies show that the first group has fewer voice quality changes, greater vocal range, and longer maximum phonation times^(5-7)^.

The practice of choral singing has many physical and psychosocial benefits for the elderly, but it also requires that the systems involved with phonation can meet the demand for vocal use for singing in order to avoid generating overload to the phonatory system, muscle compensation, and increased risk of laryngeal lesions^(8-10)^. Aging is a natural process, and even in elderly people with a healthy and vocally active lifestyle, anatomical, physiological and functional changes over time can cause vocal difficulties^(7)^.

In addition, the lack of knowledge and technique contributes to the inappropriate use of the voice and the worsening of voice-related signs and symptoms. Singers often present vocal tract dryness, fatigue, discomfort, pain, tightness in the throat, tension, vocal breaks, and voice loss, impacting the quality of life of these individuals^(11,12)^.

Therefore, the identification of situations that generate voice disadvantages for singing and related factors allows a preventive speech therapy action to be performed through more effective guidance, which in turn enhances the effects of singing for the vocal health of this population^(2,3)^. The present study sought to analyze the singing voice handicap index of elderly chorus singers and to verify its relationship with the profile, habits and health conditions of the participants.

## METHODS

This is a prospective cross-sectional study, approved by the Research Ethics Committee, under number 1.981.615. Nine amateur choirs from a city in the southern region of the country were contacted and, under the authorization of the person responsible for each of them, the research project was presented during the group rehearsal. Interested choristers were individually interviewed and invited to participate in the study by signing the Informed Consent Form (ICF). Individuals aged over 60 years and who participated in the choir for at least one year were included. Individuals who smoked and those who reported having undergone speech therapy prior to or simultaneously with the study were excluded from the study.

The elderly singers were invited to answer the questionnaire entitled “Singing Voice Handicap Index” (SVHI), developed and validated by Cohen^(13)^ and translated by Behlau and Gasparini^(14)^ The SVHI consists of 36 questions related to the impact on physical, emotional, social and economic aspects of vocal problems in singing. For each item, there are five response options “Never”, “Almost Never”, “Sometimes”, “Almost Always” and Always, scored from 0 to 4, respectively. The total score can range from 0 to 144. This considers the minimum value being the lowest voice handicap index, and the maximum value being the highest voice handicap index. The scale does not have domains and subscales. Choir singers answered the questionnaire individually before or after the rehearsal in the time necessary for each one and if they had any doubts about any question, they could ask the researchers for help. The items in the questionnaire which scored more or less were then verified.

The following data were collected through interviews: age, gender, education, vocal classification, and time in choral singing. The vocal classification had previously been determined by the conductor of each choir. The choristers were also asked about their habits and health conditions. Questions were asked about the practice of physical activity, water consumption, frequent and prolonged use of the voice, hearing difficulties, respiratory allergies, gastroesophageal reflux, throat clearing, excessive consumption of caffeinated beverages, distilled beverages, and sleep, totaling ten questions. Each question could be answered with “yes” or “no”, receiving a point for each answer unfavorable to general and vocal health, which could add up to 10 points. The data collection was carried out from April 2017 to October 2018.

The Statistical Package for the Social Sciences (SPSS) version 19.0 was used for database configuration and statistical analysis. Kolmogorov-Smirnov tests were performed to analyze the normality of variance. Continuous variables were described using the median and interquartile range, and categorical variables using absolute and relative frequency. The Mann-Whitney U test was used to compare the total score of the SVHI between the variables age, sex, and for each of the habits and health conditions. The Kruskal-Wallis test was used to compare the total score of the SVHI between levels of education, vocal classification, and time in choral singing. Spearman's correlation was also verified between the total score of the SVHI and age, time in choral singing, and scores related to habits and health conditions. Values of p<0.05 were considered statistically significant.

## RESULTS

Of the 115 choristers interviewed, five were excluded from the sample. One for being a smoker and four for having undergone speech therapy previously.

The sample had a total of 110 choristers, with a predominance of females (74.5%). The age range varied from 60 to 91 years of age, with a median of 69 (10). Most seniors were up to 75 years of age (77.3%). Higher education and/or graduate education was the most prevalent (69.1%) followed by high school (24.5%). The predominant time of choral singing was greater than 10 years (44.5%). In the composition of the sample, the singers were classified as soprano (39.1%), contralto (33.6%), tenor (18.2%), and bass (9.1%).

The singing voice handicap index had a median of 25(25) with a minimum score of 0 and a maximum score of 86. According to Chart 1, the most scored items were related to physical aspects in the use of singing voice: “I am unable to use my 'high voice'” (item 10), “My throat is dry when I sing” (item 13), “I have difficulty singing loudly” (item 21), “I have difficulty making my voice do what I want it to” (item 17), “I have difficulty controlling the breathiness in my voice” (item 19), “It takes a lot of effort to sing” (item 1) and “My ability to sing varies from day to day” (item 5). The least scored were related to economic, social, and emotional aspects: “I am worried my singing problems with cause me to lose money” (item 33), “I have to cancel performances, rehearsals or singing engagements because of my voice problem” (item 36), “I no longer want to perform or sing because of my singing difficulty” (item 8), “I feel out of the music scene because of my voice” (item 34), “When I sing, people ask what's wrong with my voice” (item 4), “I had to remove some songs from my repertoire” (item 14) and “My singing makes me feel incompetent” (item 35).

**Chart 1:** Absolute and relative frequency of the items in the Singing Voice Handicap Index

**EXPERIENCES WITH THE SINGING VOICE**	**NEVER**	**ALMOST NEVER**	**SOMETIMES**	**ALMOST ALWAYS**	**ALWAYS**
**1. It takes a lot of effort to sing**	**35 (31.8%)**	**25 (22.7%)**	**41 (37.3%)**	**6 (5.5%)**	**3 (2.7%)**
**2. My voice cracks and breaks**	**32 (29.1%)**	**35 (31.8%)**	**38 (4.5%)**	**5 (4.5%)**	**0**
3. I am frustrated by my singing	59 (53.6%)	25 (22.7%)	21 (19.1%)	5 (4.5%)	0
4. People ask “What is wrong with your voice?” when I sing	87 (79.1%)	20 (18.2%)	1 (0.9%)	2 (1.8%)	0
**5. My ability to sing varies day to day**	**33 (30.0%)**	**27 (24.5%)**	**45 (40.9%)**	**4 (3.6%)**	**1 (0.9%)**
6. My voice “gives out” on me while I am singing	42 (38.2%)	39 (35.5%)	24 (21.8%)	4 (3.6%)	1 (0.9%)
7.My singing voice upsets me	60 (54.5%)	27 (24.5%)	15 (13.6%)	6 (5.5%)	2 (1.8%)
8. My singing problems make me not want to sing/perform	97 (87.3%)	10 (9.1%)	2 (1.8%)	2 (1.8%)	0
9. I am embarrassed by my singing	76 (69.1%)	16 (14.5%)	15 (13.6%)	3 (2.7%)	0
**10. I am unable to use my “high voice”**	**28 (25.5%)**	**26 (23.3%)**	**35 (31.8%)**	**14 (12.7%)**	**7 (6.4%)**
11. I get nervous before I sing because of my singing problems	85 (77.3%)	11 (10%)	11 (10%)	2 (1.8%)	1 (0.9%)
12. My speaking voice is not normal	49 (44.5%)	34 (29.1%)	28 (25.5%)	1 (0.9%)	0
**13. My throat is dry when I sing**	**22 (20.0%)**	**28 (25.5%)**	**50 (45.5%)**	**8 (7.3%)**	**2 (1.8%)**
14. I’ve had to eliminate certain songs from my singing/performance	88 (80.0%)	17 (15.5%)	3 (2.7%)	2 (1.8%)	0
15. I have no confidence in my singing voice	52 (47.3%)	26 (23.6%)	21 (19.1%)	9 (8.2%)	2 (1.8%)
16. My singing voice is never normal	47 (42.7%)	29 (26.4%)	30 (27.3%)	3 (2.7%)	1 (0.9%)
**17. I have trouble making my voice do what I want it to**	**30 (27.3%)**	**34 (30.9%)**	**33 (30.0%)**	**11 (10%)**	**2 (1.8%)**
**18. I have to “push it” to produce my voice when singing**	**44 (40.0%)**	**28 (25.0%)**	**33 (30.0%)**	**4 (3.6%)**	**1 (0.9%)**
**19. I have trouble controlling the breathiness in my voice**	**33 (30.0%)**	**26 (23.6%)**	**41 (37.3%)**	**8 (7.3%)**	**2 (1.8%)**
20. I have trouble controlling the raspiness in my voice	44 (40%)	34 (30.9%)	24 (21.8%)	8 (7.3%)	0
**21. I have trouble singing loudly**	**36 (32.7%)**	**21 (19.1%)**	**39 (35.5%)**	**10 (9.1%)**	**4 (3.6%)**
22. I have difficulty staying on pitch when I sing	41 (37.3%)	33 (30.0%)	27 (24.5%)	8 (7.3%)	1 (0.9%)
23. I feel anxious about my singing	64 (58.2%)	24 (21.8%)	19 (17.3%)	2 (1.8%)	1 (0.9%)
24. My singing sounds forced	53 (48.2%)	30 (27.3%)	23 (20.9%)	4 (3.6%)	0
25. My speaking voice is hoarse after I sing	64 (58.2%)	25 (22.7%)	18 (16.4%0	3 (2.7%)	0
26. My voice quality is inconsistente	37 (33.6%)	40 (36.4%)	25 (22.7%)	6 (5.5%)	2 (1.8%)
27. My singing voice makes it difficult for the audience to hear me	79 (71.8%)	16 (14.5%)	9 (8.2%)	5 (4.5%)	1 (0.9%)
28. My singing makes me feel handicapped	88 (80.0%)	10 (9.1%)	8 (7.3%)	2 (1.8%)	2 (1.8%)
29. My singing voice tires easily	60 (54.5%)	27 (24.5%)	21 (19.1%)	2 (1.8%)	0
30. I feel pain, tickling, or choking when I sing	73 (66.4%)	24 (21.8%)	12 (10.9%)	1 (0.9%)	0
31. I am unsure of what will come out when I sing	52 (47.3%)	30 (27.3%)	24 (21.8%)	3 (2.7%)	1 (0.9%)
32. I feel something is missing in my life because of my inability to sing	89 (80.9%)	9 (8.2%)	9 (8.2%)	3 (2.7%)	0
33. I am worried my singing problems will cause me to lose Money	107 (97.3%)	3 (2.7%)	0	0	0
34. I feel left of of the music scene because of my singing	95 (86.4)	7 (6.4%)	6 (5.5%)	1 (0.9%)	1 (0.9%)
35. My singing makes me feel incompetente	91 (82.7%)	10 (9.1%)	6 (5.5%)	2 (1.8%)	1 (0.9%)
36. I have to cancel performances, singing engagements, rehearsals, or practices because of my singing	94 (85.5%)	14 (12.75)	1 (0.9%)	1 (0.9%)	0

The values of the questionnaire according to the characteristics of the participants can be seen in Table 1. When comparing the medians of the female vocal classifications, it was verified that the contralto choir singers presented a singing voice handicap index significantly higher than the soprano choir singers, while there was no difference when comparing the male vocal classifications.

**Table 1:** Singing voice handicap index according to the characteristics of the chorus singers

	**SVHI (n=110)**	**p-value**
	Median (IQR)	
**Age**		0.020*
60 to 75	22 (23)	
76 or higher	32 (23)	
**Gender**		0.708
Male	20 (26)	
Female	25 (25)	
**Education**		0.223
Complete elementary studies	16 (24)	
Complete high school	24 (30)	
Undergraduate and graduate degree	25.50 (25)	
**Voice classification**		
Bass	16.50 (18)	p=0.243
Tenor	22.50 (25)	
Contralto	31 (27)	p=0.023*
Soprano	24 (26)	
**Time of choral singing**		0.264
Up to three years	22 (25)	
Four to 10 years	30.54 (20)	
More than 10 years	21 (25)	

Kruskal Wallis Test; Mann-Whitney U test

* p≤0.05 statistically significant values

As for habits and health conditions, 23 (20.9%) reported not practicing any type of physical activity and 75 (68.2%) claimed to consume little water. In addition, 70 (63.6%) reported that they talk a lot, 52 (47.3%) that they slept little, 33 (30%) that they consumed caffeine in excess, and 22 (20%) distilled beverages. It was also identified that 37 (33.6%) had hearing difficulties, 47 (42.7%) had respiratory allergies, 32 (29.1%) had gastroesophageal reflux and 36 (32.7%) had frequent throat clearing. The relationship between the median of the SVHI score for each answer regarding habits and health conditions, represented by the p-value, is described in Table 2.

**Table 2:** Relationship between the SVHI scores and habits and health conditions

**Habits and health conditions**	**SVHI (n=110)**		**p-value**
	Median (IQR)		
	**Yes**	**No**	
Practices physical activity	23 (25)	33 (22)	0.094
Drinks little water	27 (24)	19 (26)	0.007*
Speaks a lot	25.50 (33)	22.50 (33)	0.458
Sleeps little	25 (19)	23.50 (30)	0.353
Excess caffeine	26 (19)	23 (29)	0.409
Distilled liquor	27 (24)	24 (27)	0.396
Hearing impairments	27 (24)	22 (26)	0.115
Respiratory allergies	25 (22)	24 (29)	0.760
Gastroesophageal Reflux	26.50 (22)	23.50 (27)	0.335
Throat clearing	29.50 (24)	23.50 (26)	0.381

Mann-Whitney U Test;

* p≤0.05 statistically significant values

Caption: IQR = Interquartile Range

There were positive correlations, although considered weak, between the total score of the SVHI and the age variable, as well as for the score of the habits and health conditions questionnaire, as can be seen in Table 3.

**Table 3:** Correlation between the Singing Voice Handicap Index and age, chorus singing time and habits questionnaire score

**Variables**	**Total SVHI score**	
	R	p
Age	0.224	0.021*
Time of choral singing	0.100	0.297
Habits score	0.250	0.008*

Spearman correlation

* p≤0.05 statistically significant values

## DISCUSSION

The choristers in this study presented an SVHI score compatible with healthy voices for singing, as found by Cohen (2007) in the validation of the instrument used^(13)^. The score was also similar to the mean normative value of a group of 729 singers healthy professionals aged between 18 and 64 years, published in a recent systematic review, who presented an SVHI of 20.35 with a confidence interval between 10.6 and 30.1^(15)^. Studies that have validated the protocol in different languages have shown that it is a valid and reliable method for identifying the individual's self-perception concerning the impact of vocal problems in singing^(16-21)^.

Items related to physical/functional problems were scored higher than those related to social/emotional aspects, results that can be compared to those of a study that evaluated 526 amateur chorus singers and found the presence of vocal symptoms but with a mild level of anxiety.^(9)^ This study used the questionnaire “Modern Singing Handicap Index” (MSHI) and specific questionnaires for vocal symptoms and to assess anxiety. MSHI, like SVHI, is adapted for singing voice, but this one specifically for modern singing. It is divided into three subscales: disability, handicap, and defect, which correspond respectively to the functional, emotional, and organic domains, which makes it possible to identify which domain the singer has the greatest disadvantage for singing. The SVHI, despite addressing these domains in its questions, does not measure them separately, that is, it does not have subscales. However, when which questions had the highest frequency was verified, it was observed that those related to physical/functional aspects were more frequent compared to the others.

A study that used the SVHI to assess 171 singers found that age is an important predictive factor for the greatest handicap in the use of singing voice^(22)^, a relation verified in the present study and which found that the SVHI score of the choir singers was significantly higher in the elderly over 75 years old compared to younger ones. These results suggest that increasing age contributes to a greater occurrence of problems in the use of singing voice, especially those related to physical aspects^(4,13,22)^.

On the other hand, the frequent habit of singing has a moderating effect on the vocal aging process. A study that evaluated 72 healthy adults found that singing helps to maintain pitch and amplitude stability, two important factors for effective communication. The authors also suggest that singing, representing muscle training, would help to maintain strength and control over vocal stability even in the presence of age-related changes^(4)^.

The practice of singing, when oriented, is shown to be a protective factor for vocal quality for both professional and amateur singers^(23)^. Although this study did not show the correlation between choir singing time and a lower rate of vocal handicap, another study highlighted the inversely proportional association in the scores of three singing protocols applied with the period of singing voice development^(11)^. This shows that voices trained for a longer time may have fewer vocal symptoms when they are well oriented. The speech therapy work is of fundamental importance for the development of the elderly's vocal and auditory perception, as it will provide vocal skills necessary for the practice of choral singing, such as directed vocal techniques and increased vocal range, observing the physiological aspects involved in the voice production of the elderly chorus singers^(4,8,20)^.

In the comparison between the vocal classifications, the study showed that there is a statistically significant difference in the total score of the SVHI, with the elderly female contraltos having a greater vocal handicap for singing than the sopranos. This fact is in agreement with other studies, in which the contralto choir singers had higher scores in the handicap for singing^(24)^ and had greater vocal fatigue^(25)^. In the present study, the most scored SVHI questions describing the main difficulties of the contralto choir singers, in addition to reaching the high notes, are related to the control of their voice and breathiness during singing, which instigates the hypothesis that these choir singers are under a greater effect of presbyphonia. It is questioned, therefore, whether the elderly contraltos are allocated in this vocal classification due to individual vocal characteristics or because they present more evident signs of the aging process in the voice, which would result in a deeper vocal quality^(3,26)^.

A positive, albeit weak, correlation was found between the questionnaire on vocal habits and health conditions and the SVHI protocol, suggesting that the more harmful habits and health conditions adverse to vocal production the individual has, the greater will be their singing voice handicap. The vocal and health habits of the choir singers are essential factors for the proper functioning of the phonatory system and for singing performance, being important items in the assessment and speech therapy intervention of these individuals^(6,27)^.

The findings suggest that the habit of drinking water was correlated with a lower vocal handicap for singing, confirming the benefit of hydration for vocal function also observed in other studies^(28,29)^, especially for the voice of elderly people who sing^(29)^. In another study, water intake was also more prevalent in elderly choristers than in non-choristers^(8)^, denoting that singing requires a higher level of hydration due to its importance for the balance of the phonatory system in emission. of the singing voice. This is a simple habit because it is accessible to most of the population, but it contributes considerably to the reduction of vocal complaints. This may be because it favors the flexibility, vibration, and lubrication of the vocal folds and, consequently, helps to maintain the biodynamic balance of the mechanisms involved in vocal production^(30)^.

In this study, no significant differences were observed in the SVHI scores associated with GER, respiratory allergies, and gender, which is in line with other research related to factors associated with a singing handicap^(22)^. However, for the variable gender, it is possible to verify in the literature, a greater vocal handicap in female singers, as well as in non-singers, is mainly related to the functional aspects of voice use^(16)^. As for GER and respiratory allergies, it is known that when present, they can impair vocal production. Our study did not detect these effects based on the SVHI score, possibly because the investigation was based on self-reported symptoms by the participants and not verified through objective examinations or medical history.

The other variables on habits and health conditions did not show a significant relationship with the SVHI score, even those known to be adverse to the voice. Most likely due to the methodology of the applied questionnaire, which addressed only “Yes” or “No” questions, not evaluating the frequency or duration of these variables in the routine of the elderly who participated in the study.

Regarding the limitations of the study, the absence of laryngeal image assessments to detect possible alterations in the structures of the vocal tract and the lack of specificity of the SVHI instrument for application in elderly populations stand out. In this sense, further studies are suggested that consider the otorhinolaryngological assessment and the construction of an instrument that identifies vocal characteristics and specific difficulties of populations of elderly choristers.

The study showed a median of 25(25) in the protocol's total score, positive associations with age and vocal classification, in addition to a positive correlation with vocal habits. We highlight the importance of using vocal self-perception instruments and vocal health questionnaires in amateur choirs, in order to map possible difficulties presented by this population, enabling a speech therapy intervention based on the choir's needs. In future studies, we suggest the adaptation of the instrument for application to elderly choristers, aiming to meet the specificities and demands involved in the activity of choral singing at this stage of life.

## CONCLUSION

The choristers in this study presented a singing voice handicap index compatible with healthy singing voices. When verifying the relationship of the SVHI score with the characteristics of the participants and with respect to habits and health conditions, it was found that the elderly choir singers over 75 years old, the contralto choir singers, and those who claimed to drink little water had higher scores of voice handicap for singing.
