# Supplementary material for: Analysis of the singing voice handicap index for elderly choristers
Source: Codas. 2022 Oct 25;34(1):e20200302. doi: 10.1590/2317-1782/20212020302 (PMC9886114; doi:10.1590/2317-1782/20212020302)
Supplement: Translation

# Translation: Análise do índice de desvantagem vocal para o canto de coristas idosos

**Authors:** Weluma Evelyn Rodrigues Moura, Camila Dalbosco Gadenz, Isadora de Oliveira Lemos, Ângelo José Gonçalves Bós, Mauriceia Cassol

**Affiliations:** 1 Departamento de Fonoaudiologia, Universidade Federal de Ciências da Saúde – UFCSPA - Porto Alegre (RS), Brasil.; 2 Departamento de Medicina, Pontifícia Universidade Católica do Rio Grande do Sul – PUCRS - Porto Alegre (RS), Brasil.

**Keywords:** Voz, Qualidade Vocal, Canto, Envelhecimento, Qualidade de Vida

## Abstract

**Objetivo:**

Analisar o índice de desvantagem vocal para o canto de coristas idosos e verificar a sua relação com perfil, hábitos e condições de saúde dos participantes.

**Método:**

Foram incluídos 110 indivíduos com 60 anos ou mais, participantes de coros amadores. Os coristas foram entrevistados a fim de verificar dados como idade, tempo de canto coral, classificação vocal e a presença de hábitos e condições de saúde adversas à produção vocal. Posteriormente, responderam o questionário “Índice de Desvantagem Vocal para o Canto (IDV-C)” que avalia a autopercepção do indivíduo em relação às experiências no uso da voz cantada.

**Resultados:**

O escore do IDV-C teve uma mediana de 25, sendo a pontuação mínima 0 e a máxima 86. Os itens mais pontuados estavam relacionados aos aspectos físicos no uso da voz cantada: “Não consigo cantar agudo” (Q10) e “Minha garganta fica seca quando canto” (Q13). Verificou-se que os idosos com mais de 75 anos apresentaram maior desvantagem vocal para o canto ao comparar com os mais jovens (p=0,020). As coristas classificadas como contralto também apresentaram maior escore do IDV-C (p=0,023), assim como os indivíduos que relataram beber pouca água (p=0,007).

**Conclusão:**

Os coristas deste estudo apresentaram um índice de desvantagem vocal para o canto compatível com vozes saudáveis para o canto. Ao verificar a relação do escore do IDV-C com as características dos participantes e a respeito dos hábitos e condições de saúde, constatou-se que os coristas idosos com mais de 75 anos, as coristas contralto e aqueles que afirmaram beber pouca água apresentaram escores maiores de desvantagem vocal para o canto.

## INTRODUÇÃO

As alterações da voz podem surgir em qualquer idade por motivos diversos, porém, no decorrer do envelhecimento há um processo natural e gradual da perda da eficiência vocal conhecido como presbifonia, tendo como sintomas comuns, a presença de soprosidade, rouquidão, alterações de *loudness*, o aumento do *pitch* para os homens e diminuição para as mulheres, instabilidade e o maior esforço durante a fonação^(1-3)^.

As alterações vocais decorrentes do envelhecimento apresentam-se, no entanto, mais sutis em indivíduos que cantam¸ uma vez que o canto frequente atua como um moderador dos efeitos negativos relacionados a esse processo^(4)^. Ao comparar as vozes de idosos cantores e não cantores, os estudos demonstram que o primeiro grupo apresenta menos alterações de qualidade vocal, maior extensão vocal e tempos máximos de fonação mais longos^(5-7)^.

A prática do canto coral apresenta muitos benefícios físicos e psicossociais ao indivíduo idoso, mas também exige que os sistemas envolvidos com a fonação consigam suprir a demanda do uso vocal para o canto para que evitem gerar sobrecargas ao aparelho fonador, compensações musculares e o aumento do risco de ocorrer lesões laríngeas^(8-10)^. O envelhecimento é um processo natural e mesmo em idosos com um estilo de vida saudável e vocalmente ativos, as modificações anatômicas, fisiológicas e funcionais ao longo do tempo, podem gerar dificuldades e desvantagens vocais^(7)^.

Além disso, a falta de conhecimento e técnica contribuem para o uso inapropriado da voz e para o agravamento dos sinais e sintomas relacionados à voz. Os cantores apresentam frequentemente ressecamento do trato vocal, fadiga, desconforto, dor, sensação de aperto na garganta, tensão, quebras vocais e perda da voz, impactando a qualidade de vida destes indivíduos^(11,12)^.

Portanto, a identificação de situações que geram desvantagens vocais para o canto e de fatores relacionados permite que seja realizada uma atuação fonoaudiológica preventiva por meio de orientações mais efetivas, que por sua vez potencializem os efeitos do canto para a saúde vocal a esta população^(2,3)^. O presente estudo buscou analisar o índice de desvantagem vocal para o canto de coristas idosos e verificar a sua relação com perfil, hábitos e condições de saúde dos participantes.

## MÉTODO

Trata-se de um estudo transversal prospectivo, aprovado pelo Comitê de Ética em Pesquisa, sob o número 1.981.615. Foram contatados nove coros amadores de uma cidade da região sul do país e sob a autorização do responsável por cada um deles, o projeto de pesquisa foi apresentado durante o ensaio dos grupos. Os coristas interessados foram entrevistados e convidados individualmente a participar da pesquisa por meio da assinatura do Termo de Consentimento Livre Esclarecido (TCLE). Foram incluídos os indivíduos com idade igual ou maior que 60 anos e que participavam do coro por pelo menos um ano. Foram excluídos da pesquisa os indivíduos tabagistas e aqueles que declararam ter realizado acompanhamento fonoaudiológico anterior ou simultâneo ao estudo.

Os idosos foram convidados a responderem o questionário intitulado “Índice Desvantagem Vocal para o Canto” (IDV-C), elaborado e validado por Cohen^(13)^ e traduzido por Behlau e Gasparini^(14)^. O IDV-C é composto por 36 questões relacionadas com o impacto nos aspectos físico, emocional, social e econômico dos problemas vocais no canto. Para cada item, há cinco opções de respostas “Nunca”, “Quase nunca”, “Às vezes”, “Quase sempre” e Sempre, pontuadas de 0 a 4, respectivamente. O escore total pode variar de 0 a 144, sendo considerado o valor mínimo, o menor índice de desvantagem vocal e o valor máximo, o maior índice de desvantagem vocal. A escala não possui domínios e subescalas. Os coristas deveriam responder o questionário individualmente antes ou após o ensaio no tempo necessário para cada um e caso tivessem dúvidas em relação à alguma questão, poderiam solicitar auxílio aos pesquisadores. Foi verificado quais foram os itens do questionário mais ou menos pontuados.

Foram coletados, por meio de entrevista, os seguintes dados: idade, sexo, escolaridade, classificação vocal e tempo de canto coral. A classificação vocal já havia sido determinada anteriormente pelo regente de cada coro. Os coristas foram questionados também a respeito dos seus hábitos e condições de saúde. Foram realizadas perguntas sobre a prática de atividade física, consumo de água, uso frequente e prolongado da voz, dificuldade auditiva, alergias respiratórias, refluxo gastroesofágico, pigarro, consumo excessivo de bebidas com cafeína, bebidas destiladas e sono, totalizando dez questões. Cada pergunta poderia ser respondida com “sim” ou “não”, recebendo um ponto a para cada resposta desfavorável à saúde geral e vocal, podendo somar 10 pontos. A coleta foi realizada no período de abril de 2017 a outubro de 2018.

Foi utilizado o *Statistical Package for the Social Sciences* (SPSS) versão 19.0 para configuração do banco de dados e análise estatística. Foram realizados os testes de Kolmogorov-Smirnov para a analisar a normalidade de variância. As variáveis contínuas foram descritas por meio da mediana e intervalo interquartil e as categóricas por meio da frequência absoluta e relativa. Foi utilizado o teste de U de Mann-Whitney para comparar o escore total do IDV-C entre as variáveis idade, sexo e para cada um dos hábitos e condições de saúde. O teste de Kruskal-Wallis foi utilizado para comparar o escore total do IDV-C entre os níveis de escolaridade, classificação vocal e tempo de canto coral. Verificou-se também a correlação de Spearman entre o escore total do IDV-C e a idade, tempo de canto coral e pontuação relacionada aos hábitos e condições de saúde. Foram considerados estatisticamente significativos os valores de p<0,05.

## RESULTADOS

Foram entrevistados 115 coristas. Destes, cinco foram excluídos: uma por ser fumante e quatro por terem realizado tratamento fonoaudiológico prévio.

A amostra teve um total de 110 coristas, com predomínio do sexo feminino (74,5%). A faixa etária variou entre 60 a 91 anos de idade, tendo como mediana 69 (10). A maioria dos idosos apresentavam até 75 anos de idade (77,3%). O grau de escolaridade de ensino superior e/ou pós-graduação foi o mais predominante (69,1%) seguido do ensino médio (24,5%). O tempo de canto coral predominante foi maior que 10 anos (44,5%). Na composição da amostra, os coristas foram classificados como soprano (39,1%), contralto (33,6%), tenor (18,2%) e baixo (9,1%).

O índice de desvantagem vocal para o canto teve uma mediana de 25(25) sendo a pontuação mínima 0 e a máxima 86. Conforme o Quadro 1, os itens mais pontuados estavam relacionados aos aspectos físicos no uso da voz cantada: “Não consigo cantar agudo” (item 10), “Minha garganta fica seca quando canto” (item 13), “Tenho dificuldade em cantar em forte intensidade” (item 21), “Tenho dificuldade em fazer o que quero com a minha voz” (item 17), “Quando eu canto, tenho dificuldade em controlar o ar na voz (soprosidade) (item 19), “Faço esforço para cantar” (item 1) e “Minha habilidade para cantar varia de um dia para o outro” (item 5). E os menos pontuados estavam relacionados aos aspectos econômicos, sociais e emocionais: “Preocupo-me com o fato de perder dinheiro por causa do meu problema de voz” (item 33), “Tenho que cancelar apresentações, ensaios ou compromissos de canto por causa do meu problema de voz” (item 36), “Eu não quero mais me apresentar ou cantar por causa de minha dificuldade no canto” (item 8), “Sinto-me fora do cenário musical por causa do meu problema de voz” (item 34), “Quando canto, as pessoas perguntam o que há de errado com a minha voz” (item 4), “Tive que retirar algumas músicas do meu repertório” (item 14) e “A dificuldade de cantar faz com que eu me sinta incompetente” (item 35).

**Quadro 1:** Frequência absoluta e relativa dos itens do Índice de Desvantagem Vocal para o Canto

**EXPERIÊNCIAS COM A VOZ CANTADA**	**NUNCA**	**QUASE NUNCA**	**ÀS VEZES**	**QUASE SEMPRE**	**SEMPRE**
**1. Faço esforço para cantar**	**35 (31,8%)**	**25 (22,7%)**	**41 (37,3%)**	**6 (5,5%)**	**3 (2,7%)**
**2. Minha voz quebra**	**32 (29,1%)**	**35 (31,8%)**	**38 (4,5%)**	**5 (4,5%)**	**0**
3. Estou frustrado com o meu canto	59 (53,6%)	25 (22,7%)	21 (19,1%)	5 (4,5%)	0
4. Quando canto, as pessoas perguntam o que há de errado com a minha voz	87 (79,1%)	20 (18,2%)	1 (0,9%)	2 (1,8%)	0
**5. Minha habilidade para cantar varia de um dia para o outro**	**33 (30,0%)**	**27 (24,5%)**	**45 (40,9%)**	**4 (3,6%)**	**1 (0,9%)**
6. Vou perdendo a voz enquanto canto	42 (38,2%)	39 (35,5%)	24 (21,8%)	4 (3,6%)	1 (0,9%)
7. Fico chateado pelos problemas que a minha voz cantada tem	60 (54,5%)	27 (24,5%)	15 (13,6%)	6 (5,5%)	2 (1,8%)
8. Eu não quero me apresentar ou cantar por causa da minha dificuldade no canto	97 (87,3%)	10 (9,1%)	2 (1,8%)	2 (1,8%)	0
9. Fico com vergonha da minha voz cantada	76 (69,1%)	16 (14,5%)	15 (13,6%)	3 (2,7%)	0
**10. Não consigo cantar agudo**	**28 (25,5%)**	**26 (23,3%)**	**35 (31,8%)**	**14 (12,7%)**	**7 (6,4%)**
11. Fico nervoso antes de cantar por causa do meu problema de voz	85 (77,3%)	11 (10%)	11 (10%)	2 (1,8%)	1 (0,9%)
12. Minha voz falada está alterada	49 (44,5%)	34 (29,1%)	28 (25,5%)	1 (0,9%)	0
**13. Minha garganta fica seca quando canto**	**22 (20,0%)**	**28 (25,5%)**	**50 (45,5%)**	**8 (7,3%)**	**2 (1,8%)**
14. Tive que tirar algumas músicas do meu repertório	88 (80,0%)	17 (15,5%)	3 (2,7%)	2 (1,8%)	0
15. Não confio em minha voz cantada	52 (47,3%)	26 (23,6%)	21 (19,1%)	9 (8,2%)	2 (1,8%)
16. Minha voz cantada está alterada	47 (42,7%)	29 (26,4%)	30 (27,3%)	3 (2,7%)	1 (0,9%)
**17. Tenho dificuldade em fazer o que quero com a minha voz**	**30 (27,3%)**	**34 (30,9%)**	**33 (30,0%)**	**11 (10%)**	**2 (1,8%)**
**18. Tenho que fazer força para encaixar a voz para cantar**	**44 (40,0%)**	**28 (25,0%)**	**33 (30,0%)**	**4 (3,6%)**	**1 (0,9%)**
**19. Quando eu canto, tenho dificuldade para controlar o ar na voz (soprosidade)**	**33 (30,0%)**	**26 (23,6%)**	**41 (37,3%)**	**8 (7,3%)**	**2 (1,8%)**
20. Quando eu canto, tenho dificuldade de controlar a aspereza na minha voz	44 (40%)	34 (30,9%)	24 (21,8%)	8 (7,3%)	0
**21. Tenho dificuldade em cantar em forte intensidade**	**36 (32,7%)**	**21 (19,1%)**	**39 (35,5%)**	**10 (9,1%)**	**4 (3,6%)**
22. Tenho dificuldade para manter o tom quando canto	41 (37,3%)	33 (30,0%)	27 (24,5%)	8 (7,3%)	1 (0,9%)
23. Sinto-me ansioso por causa do problema em minha voz cantada	64 (58,2%)	24 (21,8%)	19 (17,3%)	2 (1,8%)	1 (0,9%)
24. Meu canto soa forçado	53 (48,2%)	30 (27,3%)	23 (20,9%)	4 (3,6%)	0
25. Minha voz falada fica rouca depois que canto	64 (58,2%)	25 (22,7%)	18 (16,4%0	3 (2,7%)	0
26. A qualidade da minha voz é instável	37 (33,6%)	40 (36,4%)	25 (22,7%)	6 (5,5%)	2 (1,8%)
27. O público tem dificuldade para me ouvir cantando	79 (71,8%)	16 (14,5%)	9 (8,2%)	5 (4,5%)	1 (0,9%)
28. Sinto-me prejudicado por causa do meu problema de canto	88 (80,0%)	10 (9,1%)	8 (7,3%)	2 (1,8%)	2 (1,8%)
29. Canso-me com facilidade quando canto	60 (54,5%)	27 (24,5%)	21 (19,1%)	2 (1,8%)	0
30. Tenho dor, coceira ou engasgos quando canto	73 (66,4%)	24 (21,8%)	12 (10,9%)	1 (0,9%)	0
31. Quando canto, não tenho certeza de como a minha voz vai sair	52 (47,3%)	30 (27,3%)	24 (21,8%)	3 (2,7%)	1 (0,9%)
32. Sinto-me como se faltasse alguma coisa em minha vida por causa da dificuldade que tenho de cantar	89 (80,9%)	9 (8,2%)	9 (8,2%)	3 (2,7%)	0
33. Preocupo-me com o fato de perder dinheiro por causa do meu problema de canto	107 (97,3%)	3 (2,7%)	0	0	0
34. Sinto-me fora do cenário musical por causa do meu problema de voz	95 (86,4)	7 (6,4%)	6 (5,5%)	1 (0,9%)	1 (0,9%)
35. A dificuldade de cantar faz com que eu me sinta incompetente	91 (82,7%)	10 (9,1%)	6 (5,5%)	2 (1,8%)	1 (0,9%)
36. Tenho que cancelar apresentações, ensaios ou compromissos de canto por causa do meu problema de voz	94 (85,5%)	14 (12,75)	1 (0,9%)	1 (0,9%)	0

Os valores do questionário de acordo com as características dos participantes podem ser observados na Tabela 1. Os valores do questionário de acordo com as características dos participantes podem ser observados na Tabela 1. Ao comparar as medianas das classificações vocais femininas foi verificado que as coristas contralto apresentaram o escore de desvantagem vocal para o canto significativamente maior que as coristas soprano, enquanto não houve diferença ao comparar as classificações vocais masculinas.

**Tabela 1:** Índice de Desvantagem Vocal para o Canto de acordo com as características dos coristas

	**IDV-C (n=110)**	**Valor p**
	Mediana (IIQ)	
**Idade**		0,020^*^
60 a 75	22 (23)	
76 ou mais	32 (23)	
**Sexo**		0,708
Masculino	20 (26)	
Feminino	25 (25)	
**Escolaridade**		0,223
Até o Fundamental completo	16 (24)	
Até o Ensino Médio completo	24 (30)	
Ensino Superior e Pós-graduação	25,50 (25)	
**Classificação vocal**		
Baixo	16,50 (18)	p=0,243
Tenor	22,50 (25)	
Contralto	31 (27)	p=0,023*
Soprano	24 (26)	
**Tempo de canto coral**		0,264
Até três anos	22 (25)	
Quatro a 10 anos	30,54 (20)	
Mais de 10 anos	21 (25)	

Teste Kruskal Wallis; Teste U de Mann-Whitney

* p≤0,05 valores estatisticamente significativos

Quanto aos hábitos e condições de saúde, 23 (20,9%) relataram não praticar nenhum tipo de atividade física e 75 (68,2%) afirmaram consumir pouca água. Além disso, 70 (63,6%) informaram que falam em grande quantidade, 52 (47,3%) que dormiam pouco, 33 (30%) que consumiam bebidas com cafeína em excesso e 22 (20%) bebidas destiladas. Foi identificado também que 37 (33,6%) apresentavam dificuldade auditiva, 47 (42,7%) alergias respiratórias, 32 (29,1%) refluxo gastroesofágico e 36 (32,7%) pigarro. A relação entre a mediana do escore do IDV-C para cada resposta a respeito dos hábitos e condições de saúde, representada pelo valor p, estão descritas na Tabela 2.

**Tabela 2:** Relação entre os escore do IDV-C e hábitos e condições de saúde

**Hábitos e condições de saúde**	**IDV-C (n=110)**		**Valor p**
	Mediana (IIQ)		
	**Sim**	**Não**	
Pratica atividade física	23 (25)	33 (22)	0,094
Bebe pouca água	27 (24)	19 (26)	0,007*
Fala em grande quantidade	25,50 (33)	22,50 (33)	0,458
Dorme pouco	25 (19)	23,50 (30)	0,353
Cafeína em excesso	26 (19)	23 (29)	0,409
Bebidas destiladas	27 (24)	24 (27)	0,396
Dificuldade auditiva	27 (24)	22 (26)	0,115
Alergias respiratórias	25 (22)	24 (29)	0,760
Refluxo gastroesofágico	26,50 (22)	23,50 (27)	0,335
Pigarro	29,50 (24)	23,50 (26)	0,381

Teste U de Mann-Whitney;

* p≤0,05 valores estatisticamente significativos

Legenda: IIQ = Intervalo Interquartil

Houve correlações positivas, embora consideradas fracas, entre o escore total do IDV-C e a variável idade, bem como para a pontuação do questionário de hábitos e condições de saúde como pode ser observada na Tabela 3.

**Tabela 3:** Correlação entre o Índice de Desvantagem Vocal para o Canto e a idade, tempo de canto coral e pontuação do questionário de hábitos

**Variáveis**	**Escore total IDV-C**	
	r	p
Idade	0,224	0,021^*^
Tempo de canto coral	0,100	0,297
Pontuação de hábitos	0,250	0,008*

Correlação de Spearman

* p≤0,05 valores estatisticamente significativos

## DISCUSSÃO

Os coristas deste estudo apresentaram um escore de IDV-C compatível com vozes saudáveis para o canto, como encontrado por Cohen (2007) na validação do instrumento utilizado^(13)^. O escore também foi semelhante ao valor normativo médio de um grupo de 729 cantores profissionais saudáveis com idade entre 18 e 64 anos, publicado em uma revisão sistemática recente, que apresentaram um IDV-C de 20,35 com intervalo de confiança entre 10.6 e 30.1^(15)^. Estudos que validaram o protocolo em diversos idiomas demonstraram que é um método válido e confiável na identificação da autopercepção do indivíduo em relação ao impacto dos problemas vocais no canto^(16-21)^.

Os itens relacionados a problemas físicos/funcionais foram mais pontuados do que os relacionados aos aspectos sociais/emocionais, resultados que podem ser comparados aos de um estudo que avaliou 526 coristas amadores e constatou a presença de sintomas vocais, mas com um índice leve de ansiedade^(9)^. Este estudo utilizou o questionário “Índice de Desvantagem para o Canto Moderno” (IDCM) e questionários específicos para os sintomas vocais e para avaliar a ansiedade. O IDCM, assim como o IDVC, é adaptado para a voz cantada, mas este especificamente para o canto moderno. É dividido em três subescalas: incapacidade, desvantagem e defeito, que correspondem respectivamente aos domínios funcional, emocional e orgânico, o que possibilita identificar qual o domínio que o cantor apresenta maior desvantagem para o canto. O IDVC, apesar de abordar os referidos domínios em suas questões, não as mensura separadamente, ou seja, não apresenta subescalas. Entretanto, quando foi verificado quais questões obtiveram maior frequência, observou-se que aquelas relacionadas aos aspectos físicos/funcionais foram mais frequentes comparadas às demais.

Um estudo que utilizou o IDV-C para avaliar 171 cantores verificou que a idade é um fator preditivo importante para a maior desvantagem no uso da voz cantada^(22)^, relação verificada no presente estudo e que constatou que o escore do IDV-C dos coristas foi significativamente maior nos idosos com mais de 75 anos comparado aos mais jovens. Estes resultados sugerem que o aumento da idade contribui para a maior ocorrência de problemas no uso da voz cantada, especialmente aqueles relacionados aos aspectos físicos^(4,13,22)^.

Por outro lado, o hábito frequente de cantar apresenta um efeito moderador do processo de envelhecimento vocal. Um estudo que avaliou 72 adultos saudáveis verificou que o canto auxilia na manutenção da estabilidade de *pitch* e de amplitude, dois fatores importantes para a efetividade da comunicação. Os autores sugerem ainda que o canto, representando um treino muscular, ajudaria a manter a força e controle sobre a estabilidade vocal mesmo na presença das alterações decorrentes da idade^(4)^.

A prática do canto quando orientada mostra-se ser um fator de proteção da qualidade vocal tanto para cantores profissionais, quanto amadores^(23)^. Embora este estudo não tenha evidenciado a correlação do tempo de canto coral ao menor índice de desvantagem vocal, outra pesquisa destacou a associação inversamente proporcional nos escores de três protocolos de canto aplicados com o período de desenvolvimento da voz cantada^(11)^, evidenciando que as vozes treinadas por mais tempo podem ter menos sintomas vocais quando são bem orientadas. É de fundamental importância o trabalho fonoaudiológico para o desenvolvimento da percepção vocal e auditiva dos idoso, visto que irá proporcionar habilidades vocais necessárias para a prática do canto coral, como técnicas vocais direcionadas e aumento da tessitura vocal, observando os aspectos fisiológicos envolvidos na produção da voz do idoso corista^(4,8,20)^.

Na comparação entre as classificações vocais, o estudo mostrou que há uma diferença estatisticamente significativa do escore total do IDV-C, tendo as idosas contraltos apresentado maior desvantagem vocal para o canto do que as sopranos. Esse fato concorda com outros estudos, nos quais as coristas contralto tiveram maiores escores na desvantagem para o canto^(24)^ e apresentaram maior cansaço vocal^(25)^. No presente estudo, as perguntas do IDV-C mais pontuadas e que descrevem as principais dificuldades das coristas contralto, além do alcance às notas agudas, estão relacionadas ao controle da própria voz e da soprosidade durante o canto, o que instiga a hipótese de que estas coristas estejam sob maior efeito da presbifonia. Questiona-se, portanto, se as contraltos idosas estão alocadas nessa classificação vocal devido às características vocais individuais ou por apresentarem sinais mais evidentes do processo de envelhecimento na voz, o qual acarretaria numa qualidade vocal mais grave^(3,26)^.

Foi encontrada uma correlação positiva, embora fraca, entre o questionário de hábitos vocais e condições de saúde e o protocolo IDV-C, sugerindo que quanto mais hábitos nocivos e condições de saúde adversas à produção vocal o indivíduo possui, maior será a sua desvantagem vocal para o canto. Os hábitos vocais e de saúde dos coristas são fatores essenciais para o adequado funcionamento do sistema fonatório e para a performance no canto, sendo itens importantes na avaliação e intervenção fonoaudiológica destes indivíduos^(6,27)^.

Os achados sugerem que o hábito de tomar água esteve correlacionado à menor desvantagem vocal para o canto, confirmando o benefício da hidratação para a função vocal observado também em outros estudos^(28,29)^, especialmente para a voz do idoso que canta^(29)^. Em outro estudo a ingestão de água também mostrou-se mais prevalente em idosas coristas do que nas não coristas^(8)^, denotando que a prática do canto exige um maior nível de hidratação devido à sua importância para o equilíbrio do sistema fonatório na emissão da voz cantada. Este é um hábito simples por ser acessível a maioria da população, mas que contribui consideravelmente para a diminuição das queixas vocais, uma vez que favorece a flexibilidade, vibração e lubrificação das pregas vocais e, por consequência, ajuda a manter o equilíbrio biodinâmico dos mecanismos envolvidos na produção vocal^(30)^.

Nesse estudo não foram observadas diferenças significativas nos escores do IDV-C associadas ao RGE, alergias respiratórias e sexo, estando em consonância com outra pesquisa relacionada a fatores associados a desvantagem para o canto^(22)^. No entanto para a variável sexo é possível verificar na literatura uma maior desvantagem vocal em mulheres cantoras, bem como em não cantoras relacionado, principalmente, aos aspectos funcionais do uso da voz^(16)^. Quanto ao RGE e alergias respiratórias, sabe-se que quando presentes estes podem prejudicar a produção vocal. Nosso estudo não detectou esses efeitos a partir do escore do IDV-C, possivelmente, porque a investigação foi realizada com base nos sintomas auto-referidos pelos participantes e não verificados através de exames objetivos ou histórico médico.

As demais variáveis sobre hábitos e condições de saúde não apresentaram relação significativa com a pontuação do IDV-C, mesmo as conhecidas adversas à voz. Provavelmente pela metodologia do questionário aplicado, que abordou questões somente de “Sim” ou “Não”, não avaliando a frequência e nem duração dessas variáveis na rotina dos idosos que participaram da pesquisa.

Com relação às limitações do estudo, destacam-se a ausência de avaliação de imagem laríngea para a detecção de possíveis alterações de estruturas do trato vocal e a falta de especificidade do instrumento IDV-C para a aplicação em populações idosas. Neste sentido, sugere-se mais pesquisas que considerem a avaliação otorrinolaringológica e a construção de um instrumento que identifique características vocais e dificuldades específicas de populações de coristas idosos.

O estudo demonstrou uma mediana de 25(25) na pontuação total do protocolo, associações positivas com idade e classificação vocal, além de uma correlação positiva com hábitos vocais. Destacamos a importância do uso de instrumentos de autopercepção vocal e de questionários de saúde vocal em coros amadores, a fim de mapear possíveis dificuldades apresentadas por esta população, possibilitando uma intervenção fonoaudiológica baseada nas necessidades do coro. Em estudos futuros, sugerimos a adaptação do instrumento para a aplicação em coristas idosos, visando atender às particularidades e às demandas envolvidas na atividade do canto coral nesta fase da vida.

## CONCLUSÃO

Os coristas deste estudo apresentaram um índice de desvantagem vocal para o canto compatível com vozes saudáveis para o canto. Ao verificar a relação do escore do IDV-C com as características dos participantes e a respeito dos hábitos e condições de saúde, constatou-se que os coristas idosos com mais de 75 anos, as coristas contralto e aqueles que afirmaram beber pouca água apresentaram escores maiores de desvantagem vocal para o canto.
